# Characterization of olfactomedin 4+ cells in prostate and urethral-tube epithelium during murine postnatal development and in adult mice

**DOI:** 10.1038/s41598-023-37320-9

**Published:** 2023-06-25

**Authors:** Hongzhen Li, Vijender Chaitankar, Lena Cui, Weiping Chen, Kyung Chin, Jianqiong Zhu, Wenli Liu, Griffin P. Rodgers

**Affiliations:** 1 Molecular and Clinical Hematology Branch, National Heart, Lung, and Blood Institute, National Institutes of Health, Bldg. 10, Room 9N119, 9000 Rockville Pike, Bethesda, MD 20892 USA; 2 Bioinformatics and Systems Biology Core, National Heart, Lung, and Blood Institute, National Institutes of Health, Bethesda, MD 20892 USA; 3 Genomics Core, National Institute of Diabetes and Digestive and Kidney Diseases, National Institutes of Health, Bethesda, MD 20892 USA

**Keywords:** Organogenesis, Cell biology, Developmental biology

## Abstract

Olfactomedin4 (*Olfm4*) is expressed in normal mouse prostate. However, Olfm4+ cells in the murine prostate have not been well characterized. In this study, we generated an *Olfm4*^*eGFP*^ reporter mouse line with C57BL/6 mice and investigated the distribution of Olfm4/eGFP-expressing cells during postnatal development from P1, P7, P14, P20, P42, P56 to adult male mouse prostate and urethral tube. We observed Olfm4/eGFP expression in urogenital and prostatic epithelial cells during early postnatal development, which persisted into adulthood in urethral-tube and anterior-prostate (AP) epithelium. We found Olfm4+ cells are E-cadherin+/CD44+/Foxa1+ and some of subpopulation are Ck8+/Ck5+/Sca-1-/Ck4-/Syn- in the adult mouse AP epithelium. Functional studies of single-cell preparations of Olfm4/eGFP-expressing cells isolated from adult *Olfm4*^*eGFP*^ mouse prostate demonstrated that Olfm4+ cells can grow and form colonies, spheres, or organoids in culture. Bioinformatic analysis of Olfm4+ cells using single-cell RNA sequencing meta data in adult mouse urethra (GSE145865) identified upregulation of genes related to cell and tissue migration and development, as well as upregulation of xenobiotic metabolism signaling pathways. In conclusion, *Olfm4*^*eGFP*^ mouse is a novel model to further study *Olfm4’*s biological functions and Olfm4+ cells may contribute importantly to cellular processes supporting development and homeostasis of the epithelium in murine prostate and urethral tube.

## Introduction

The olfactomedin 4 (*OLFM4*) gene is a member of the olfactomedin gene family, which is conserved between human and mouse and plays important roles in development and disease^1–4^. OLFM4 mRNA expression has been detected in normal human prostate tissues, primary-culture normal human prostate epithelial cells, and immortalized normal human prostate epithelial cells (RWPE1 cell line)^5–7^. Recently, we have demonstrated that *OLFM4* is expressed in Club and Hillock cells in normal human adult prostate, as well as in multiple stem/progenitor-like cell populations found in RWPE1 cultures^8^.

The murine *Olfm4* gene (also called PU.1 difference product 4 [pDP4]) is a PU.1 transcription factor target gene and shares 85% cDNA similarity with the human *OLFM4* gene^9^. It is located on murine chromosome 14, contains 5 exons, and encodes a secreted 57-kDa glycoprotein with an olfactomedin domain^9^. Studies using a conventional *Olfm4* knockout mouse model have demonstrated that *Olfm4* plays critical roles in innate immunity, inflammation, cancers, and obesity^10–14^. We have previously detected Olfm4 mRNA expression in *Olfm4* wild-type mouse prostate and found that aging *Olfm4* knockout mice sporadically developed prostatic neoplasia^12^. However, the distribution and function of Olfm4+ cells in the adult mouse prostate and the expression pattern during postnatal development have not been well understood.

In this study, we characterized Olfm4+ cells during murine prostate and urethral-tube postnatal development, as well as in adult mouse prostate and urethral-tube. We found that Olfm4 was expressed in urogenital and prostatic epithelial cells in early postnatal development and persisted in urethral-tube and anterior-prostate epithelium both in later postnatal development and in adult mice. Functional analyses in vitro demonstrated that Olfm4+ cells isolated from adult mouse prostate could grow and form colonies, spheres, or organoids in 2-dimensional (2D) and 3-dimensional (3D) cultures. Olfm4+ cells in the urethral-tube epithelium were identified as luminal epithelial cells by analyses of single-cell RNA sequencing data (from a previously generated dataset for adult mouse urethra [GSE145865]). Gene Ontology and Ingenuity Pathway analyses of single-cell RNA sequencing data for Olfm4+ urethral luminal epithelial cells identified upregulation of genes related to cell and tissue migration and development, as well as upregulation of 4 xenobiotic metabolism signaling pathways. Our results suggest that Olfm4+ cells may contribute importantly to cellular processes supporting development and homeostasis of the epithelium in murine prostate and urethral tube. The *Olfm4*^*eGFP*^ mouse is a novel model to further study Olfm4’s biological functions in murine tissues and could be used to further understand the role of OLFM4 during normal human development and disease progression.

## Results

### Olfm4 is strongly expressed in epithelial cells in the anterior prostate and urethral tube of adult mice

**Figure 1:**
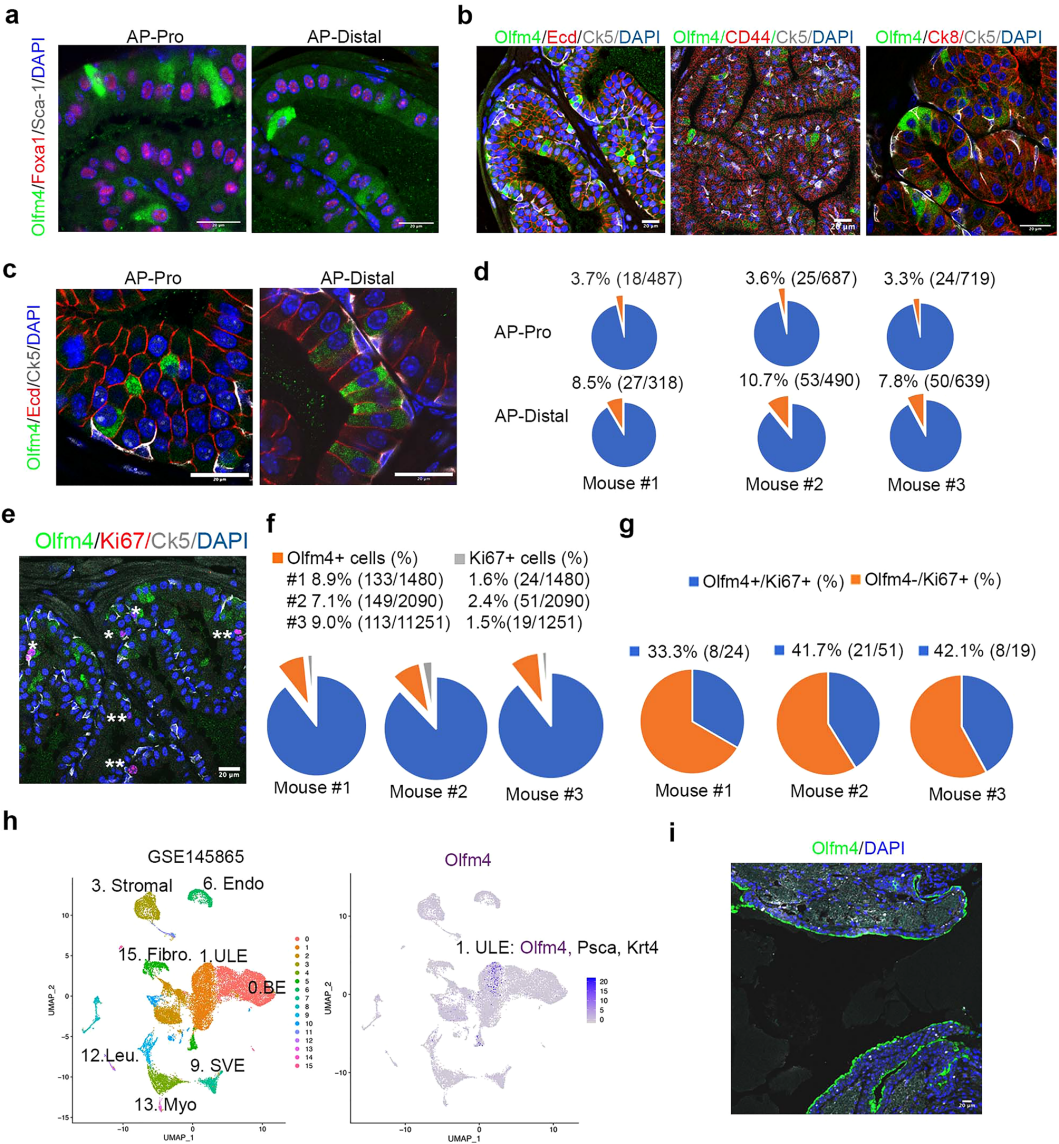
Olfm4 is strongly expressed in epithelial cells of anterior prostate and urethral tube of adult mice. (**a**) Representative merged images from triple-color immunofluorescent staining in anterior prostate of adult mice with Olfm4, Foxa1, and Sca-1 antibodies. AP-Pro, proximal anterior prostate; AP-Distal, distal anterior prostate. Scale bars: 20 μm. Blue represents DAPI nuclear staining. (**b**) Representative merged images from triple-color immunofluorescent staining in anterior prostate of adult mice with Olfm4, Ck5, and E-cadherin (Ecd), CD44, and Ck8 antibodies. Scale bars: 20 μm. Blue represents DAPI nuclear staining. (**c**) Representative merged images from triple-color immunofluorescent staining in anterior prostate of adult mice with Olfm4, E-cadherin (Ecd) and Ck5 antibodies. Scale bars: 20 μm. Blue represents DAPI nuclear staining. (**d**) Quantitation of the Olfm4+ cells within the proximal and distal AP of adult mice from 1C. Data were obtained from analysis of single-color images from immunofluorescent staining with Olfm4 antibody using the 3D objects counter function (Image J) from a total of 5–10 images for each individual mouse. (**e**) Representative merged images from triple-color immunofluorescent staining in AP of *Olfm4* wild-type adult mice with Olfm4, Ki67, and Ck5 antibodies. Scale bars: 20 μm. Blue represents DAPI nuclear staining. One asterisk indicates Olfm4+/Ki67+ cells; two asterisks indicate Olfm4-/Ki67+ cells. (**f**) Quantitation of the Olfm4+ or Ki67+ cells within the AP of *Olfm4* wild-type adult mice. (**g**) Quantitation of Olfm4 expression in Ki67+ cells within the AP of *Olfm4* wild-type adult mice. (**h**) UMAP plots of integrated data from single-cell RNA sequencing of the adult mouse urethra GSE145865 dataset. Left panel shows 16 clusters of total urethral epithelium cells; right panel shows Olfm4-expressing cells (purple color) in cluster 1, urethral luminal epithelial cells (ULE). (**i**) Representative single-color image from immunofluorescent staining in urethral epithelium of lower urinary-tract sagittal sections of adult mice with Olfm4 antibody. Scale bar: 20 μm. Blue represents DAPI nuclear staining.

To investigate Olfm4-expressing cells in adult murine prostate, we performed immunohistochemical staining with an Olfm4 rabbit monoclonal antibody and found that cells expressing Olfm4 protein were distributed throughout the anterior lobe of *Olfm4* wild-type adult mouse prostate (see Supplementary Fig. S1 online). In contrast, no staining was observed in the anterior lobe of *Olfm4* knockout adult mouse prostate, indicating that the antibody specifically recognizes Olfm4 protein (see Supplementary Fig. S1 online). No Olfm4 staining of cells was observed in the ventral, dorsal, and lateral lobes of *Olfm4* wild-type adult mouse prostate (see Supplementary Fig. S1 online). Olfm4-expressing cells were detected in proximal, internal, and distal regions of *Olfm4* wild-type adult mouse anterior prostate (AP) (Fig. 1a–c and see Supplementary Fig. S1 online). Olfm4+ cells were also observed residing in the luminal epithelium of urethral tubes, but not in the epithelium of periurethral tubes, ampullary gland, seminal vesicles, or bladder of *Olfm4* wild-type adult mice (see Supplementary Fig. S1 online).

To further identify the Olfm4+ cells observed in the AP epithelium, we performed immunofluorescent staining for several different cell markers on adult mouse prostate tissues. Olfm4+ cells co-expressed Foxa1, but not Sca-1, in proximal and distal regions of AP (Fig. 1a). Foxa1 is a transcription factor that is highly expressed in the nucleus in prostate epithelial cells throughout development and in adult murine prostate^15,16^, and serves as a pioneer factor that guides the binding of chromatin for androgen receptor during prostate development^17^. Olfm4+ cells in AP tissue also co-expressed E-cadherin (Fig. 1b,c), and CD44 (Fig. 1b). Interestingly, a subpopulation of Olfm4+ cells co-expressed both Ck8, a luminal cell marker, and Ck5, a basal cell marker (Fig. 1b). Olfm4+ cells did not co-express either Ck4, or synaptophysin (Syn), a neuroendocrine cell marker in the distal AP epithelium of adult mice (see Supplementary Fig. S1 online). Quantitation of Olfm4 expression in adult mouse AP indicated that 3.5 ± 0.2% (mean ± SD) of cells in the proximal AP were Olfm4+ and 9.0 ± 1.5% of cells in the distal AP were Olfm4+ (Fig. 1c,d). Quantitation of Ki67 and Olfm4 expression in adult mouse AP indicated that 1.8 ± 0.5% (n = 3) (mean ± SD) of Ki67+ cells in total counted AP distal epithelial cells and that 38.8 ± 4.8% (n = 3) (mean ± SD) of cells that stain positively for Ki67, an indicator for proliferative cells, were also Olfm4+ (Fig. 1e–g). Taken together, we found that higher populations of Olfm4+ cells were distributed in the distal region of adult mouse AP epithelium. The Olfm4+ cells were characterized as E-cadherin+/CD44+/Foxa1+ epithelial cells and with some of the Olfm4+ cells being a subpopulation Ck8+/Ck5+/Sca-1-/Ck4-/Syn- cells as well as Olfm4+/Ki67+ proliferative cells in the adult mouse AP epithelium.

Because we observed that Olfm4 is highly expressed in the epithelium of urethral tube of adult mice, we sought to further identify the Olfm4-expressing cells by using single-cell RNA sequencing data previously generated from adult mouse urethra (GSE145865)^18^. We analyzed cell data for gene-expression signatures using Uniform Manifold Approximation and Projection (UMAP) software. Analysis of differentially expressed genes confirmed 3 previously classified epithelial clusters (BE: Basal epithelial cells; ULE: Urethral luminal epithelial cells; SVE: Seminal vesicle epithelial cells) and Endothelial epithelial cells (END) as well as 4 stromal clusters (leukocyte: Leu; stromal, fibroblast: Fibro.; and smooth muscle: Myo.) within 16 clusters (Fig. 1e). Higher populations of Olfm4-expressing cells were observed in cluster 1, urethral luminal epithelial cells (ULE), with the Olfm4-expressing cells in cluster 1 co-expressing Psca and Krt4, a marker for urethral luminal epithelial cells^18^ (Fig. 1e). Next, we performed immunofluorescent staining with Olfm4 antibody and confirmed expression of Olfm4 in the luminal urethral epithelium of lower urinary-tract sagittal sections (Fig. 1f).

### Generation and evaluation of ***Olfm4***^***eGFP***^ reporter mice

**Figure 2:**
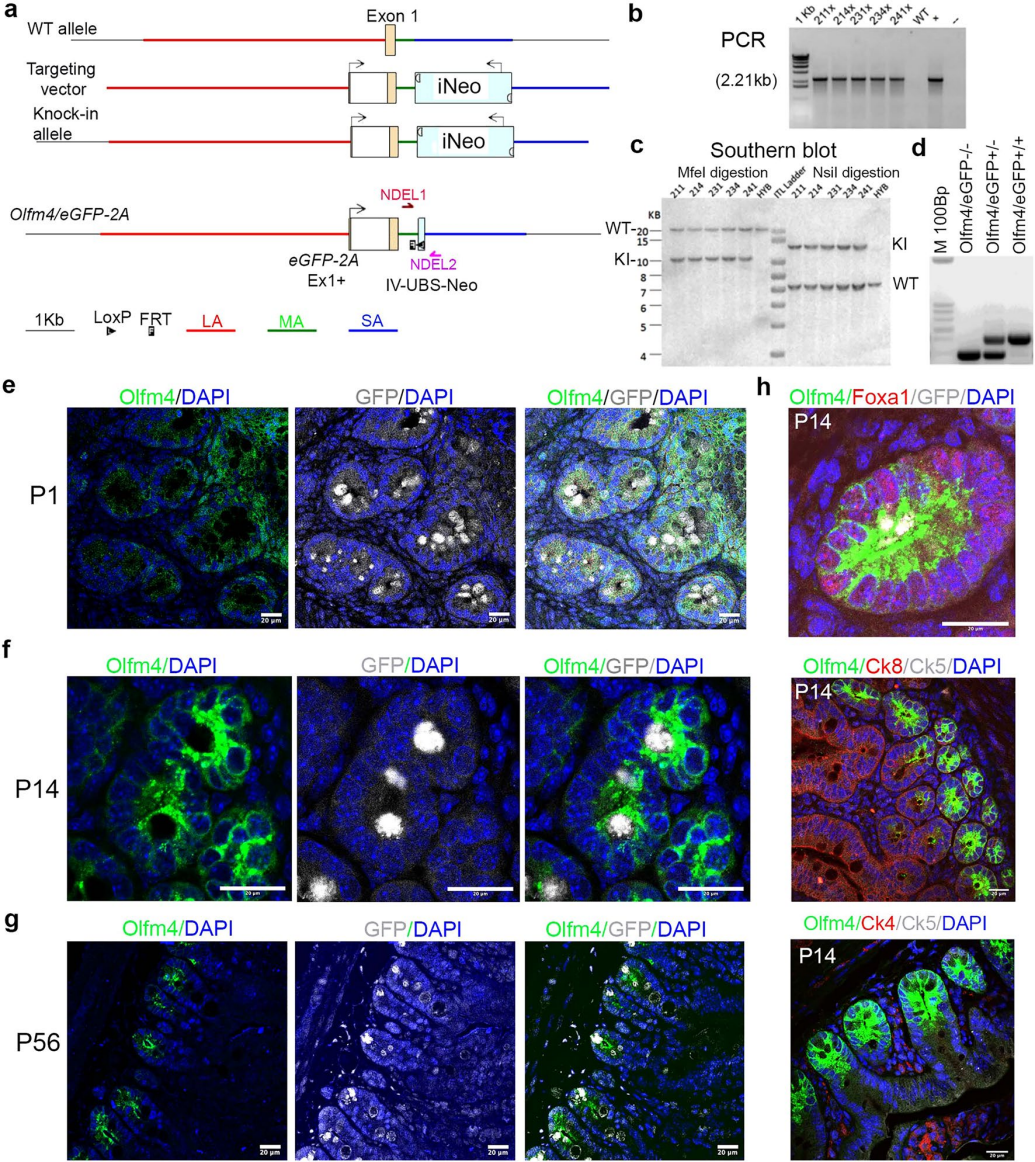
Generation and evaluation of an *Olfm4*^*eGFP*^ reporter mouse strain. (**a**) Schematic representation of the mouse *Olfm4* locus (chr14: 79,998,501–80,000,501) target vector. The region was designed such that the long-homology arm (LA) extends ~ 5.04 kb 5′ to the GFP sequence, and the short-homology arm (SA) extends 2.03 kb 3′ to the FRT-flanked Neo cassette. The *eGFP-2A* coding sequence is fused right after the endogenous ATG initiation site in exon 1. The FRT-flanked Neo cassette is inserted 397 bp downstream of exon 1. MA, middle-homology arm; WT, wild-type. (**b**) PCR analysis for identifying recombinant expanded surviving clones of BA1 (129/SvEv x C57BL/6) ES cells after selection with G418 antibiotic. PCR product size: 2.21 kb. An x denotes expanded clones. DNA from an individual clone (before reconfirmation) was used as a positive control (+). No DNA (−) and wild-type DNA (WT) were used as negative controls. (**c**) Southern-blotting analysis for confirming correct homologous recombination in positive clones identified by PCR. The expected sizes for the wild-type (WT) allele are 22.5 kb and 7.4 kb; the expected sizes for the recombined (KI) allele are 10.499 and 13.062 kb. HYB, DNA from C57BL/6 (B6), 129/SvEv (129), and BA1 (129/SvEv x C57BL/6) (Hybrid) mouse strains were used as wild-type controls. ITL Ladder, 4–20 kb. (**d**) Representative PCR genotyping of wild-type, heterozygous, and homozygous mice. Wild-type allele = 289 bp, knock-in allele = 426 bp. M 100Bp, 100-bp ladder. (**e**–**g**) Representative single-color or merged images from double-color immunofluorescent staining in small-intestine tissues of P1 (**e**), P14 (**f**), or P56 (**g**) *Olfm4*^*eGFP*^ mice with Olfm4 and GFP (ab13970, Abcam) antibodies. Scale bars: 20 μm. Blue represents DAPI nuclear staining. (**h**) Representative merged images from triple-color immunofluorescent staining in small-intestine tissues of P14 or P42 *Olfm4*^*eGFP*^ mice with Olfm4, Foxa1, and GFP (top panel), Olfm4, Ck8, and Ck5 (middle panel), or Olfm4, Ck4, and Ck5 (bottom panel) antibodies. Scale bars: 20 μm. Blue represents DAPI nuclear staining.

To further study Olfm4+ cells in mouse prostate, we generated an *Olfm4*^*eGFP*^ mouse line by constructing a targeting cassette within the enhanced GFP and 2 peptide (*eGFP-2A*) gene (Fig. 2a). This strategy allows co-expression of *Olfm4* and *eGFP* under *Olfm4* gene promoter regulation in cells. A targeting vector was constructed, transfected into BA1 (129/SvEv x C57BL/6) embryonic stem (ES) cells, and surviving clones selected. PCR analysis identified recombinant ES clones (Fig. 2b). Southern-blotting analysis confirmed correct homologous recombination in ES cell clones (Fig. 2c). Targeted ES cells were injected into blastocysts of C57BL/6 mice; PCR genotyping indicated that multiple chimeric mice were produced (Fig. 2d). Heterozygous and homozygous mice were retrieved at the expected Mendelian ratios at birth. Adult *Olfm4*^*eGFP*^ mice showed no obvious abnormalities and displayed a lifespan and fertility comparable to those of wild-type littermates (data not shown).

To confirm that eGFP protein expression in our *Olfm4*^*eGFP*^ mice could accurately report *Olfm4* expression, we examined the expression of eGFP in several tissues collected from *Olfm4*^*eGFP*^ mice. Because we previously found that *Olfm4* is expressed in murine bone marrow, small intestine, and prostate^1^, we analyzed these tissues. As expected, eGFP protein was expressed in myeloid progenitor cells and neutrophil cells in bone marrow from adult mice (see Supplementary Fig. S2 online). Immunohistochemical staining with GFP or Olfm4 antibodies confirmed that these cells express either GFP or Olfm4 (see Supplementary Fig. S2 online). Because Olfm4 mRNA is highly expressed in the small intestine, we further analyzed Olfm4 and eGFP protein expression in intestine tissue of *Olfm4*^*eGFP*^ mice as an additional confirmation. Olfm4/eGFP were highly expressed in the bottom of intestinal crypts of adult *Olfm4*^*eGFP*^ but no expression of Olfm4 was observed in intestinal tissue of *Olfm4* knockout mice (see Supplementary Fig. S2 online). Furthermore, Olfm4/eGFP was highly expressed in Olfm4+ intestinal cells in postnatal day (P) 1, P14, and P56 *Olfm4*^*eGFP*^ mice (Fig. 2e–h). These results confirmed that eGFP expression faithfully represents *Olfm4* expression in cell populations in the *Olfm4*^*eGFP*^ reporter mouse line.

### Olfm4/eGFP is expressed during early postnatal development of murine prostatic, ampullary-gland, seminal-vesicle and urethral epithelium

**Figure 3:**
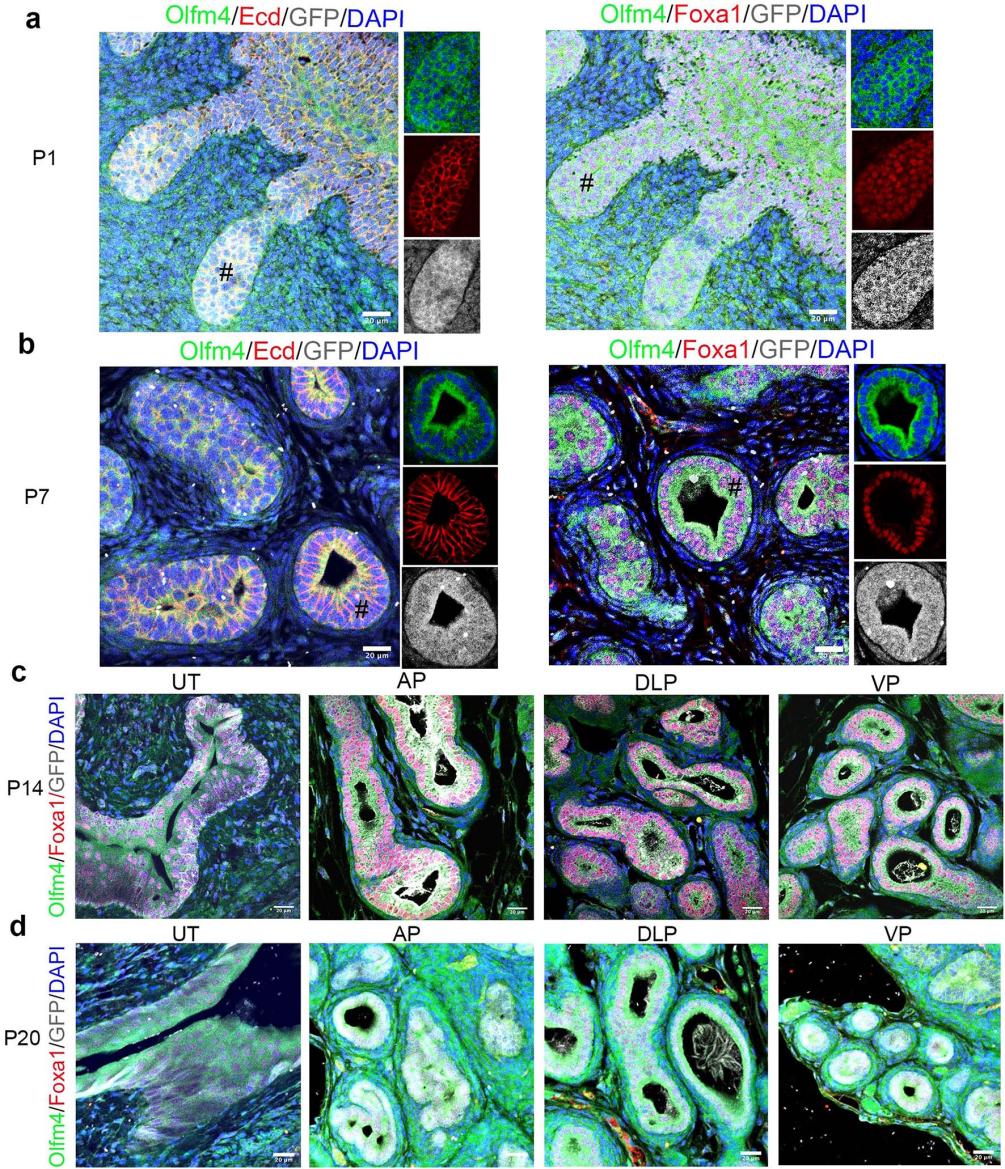
Olfm4/eGFP is expressed during early postnatal development of *Olfm4*^*eGFP*^ mouse prostate and urethral epithelium. (**a**, **b**) Representative merged and single-color images from triple-color immunofluorescent staining in P1 (**a**) or P7 (**b**) prostate tissues of *Olfm4*^*eGFP*^ mice with Olfm4, E-cadherin, and GFP (ab13970, Abcam) antibodies (left panels) or with Olfm4, Foxa1, and GFP (ab13970, Abcam) antibodies (right panels). Ecd, E-cadherin. Scale bars: 20 μm. Blue represents DAPI nuclear staining. # indicates the region shown in the single-color staining micrographs. (**c, d**) Representative merged images from triple-color immunofluorescent staining in P14 (**c**) or P20 (**d**) urethral-tube or prostate tissues of *Olfm4*^*eGFP*^ mice with Olfm4, Foxa1, and GFP (ab13970, Abcam) antibodies. UT, urethral tubes; AP, anterior prostate; DLP, dorsolateral prostate; VP, ventral prostate. Scale bars: 20 μm. Blue represents DAPI nuclear staining.

To investigate Olfm4 expression in mouse prostate during postnatal development, we performed immunofluorescent staining in *Olfm4*^*eGFP*^ mouse prostate tissues with antibodies for Olfm4, GFP, and E-cadherin or Foxa1. In P1 mouse prostate tissue, Olfm4/eGFP was expressed within E-cadherin+ and Foxa1+ prostatic-bud epithelial cells^19^ (Fig. 3a). Olfm4/eGFP, Foxa1, and E-cadherin were expressed in basal and luminal cells of prostatic acini at P7 (Fig. 3b). Olfm4/eGFP co-expression with Foxa1 was consistently observed in epithelial cells of urethral tubes, as well as in epithelial cells of anterior, dorsolateral, and ventral prostate, at P14 (Fig. 3c) and P20 (Fig. 3d). Olfm4 was co-expressed with Ck8, Ck5, and Foxa1 in ampullary-gland epithelium and seminal-vesicle epithelium at P14 (see Supplementary Fig. S3 online). These data indicates that Olfm4 is extensively expressed during early postnatal development in these epithelial cells.

### Olfm4+ epithelial cells persist during late postnatal development of murine urethral tubes, anterior prostate

**Figure 4:**
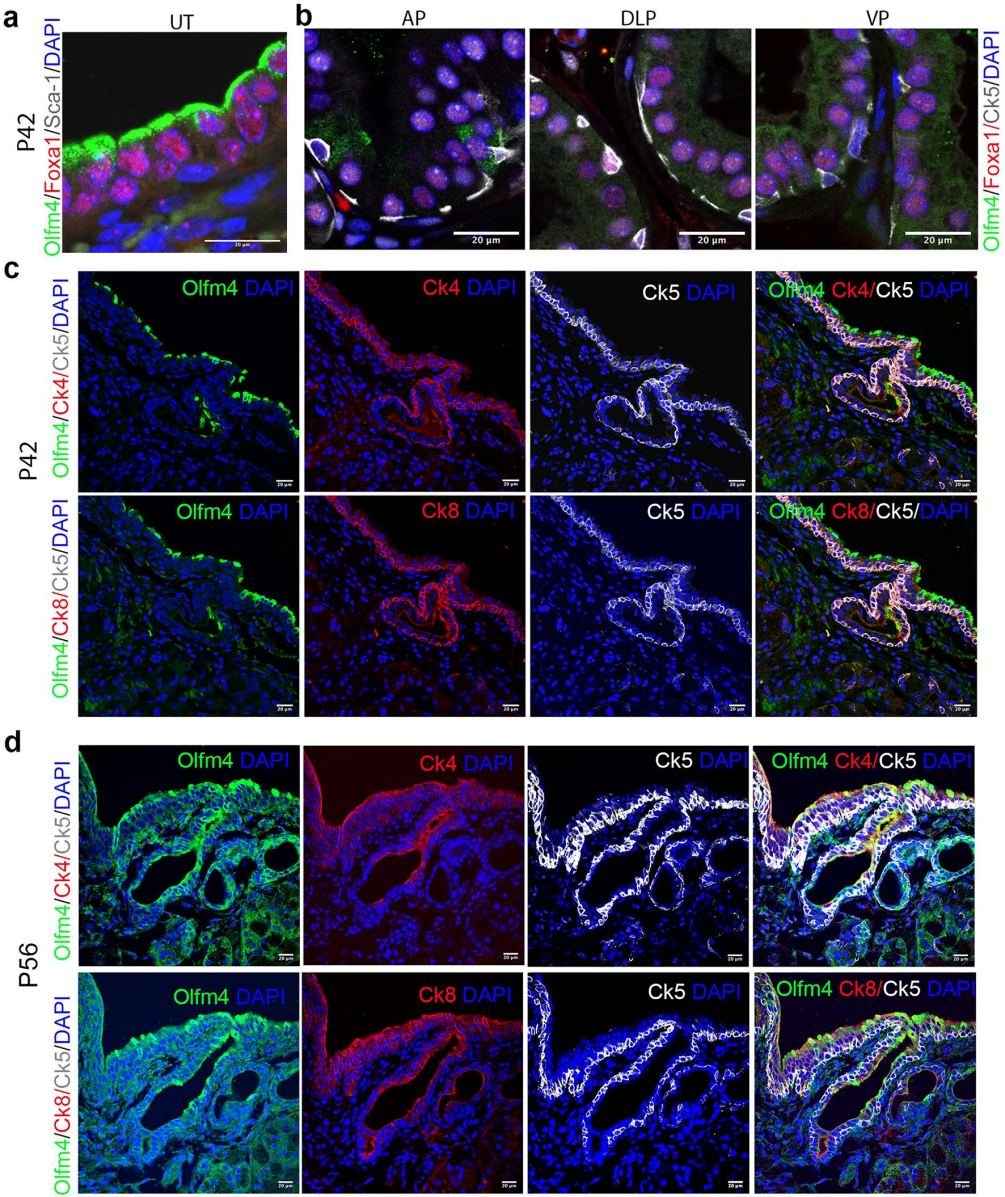
Olfm4+ cells persist during late postnatal development of *Olfm4*^*eGFP*^ mouse anterior prostate and urethral epithelium. (**a**, **b**) Representative merged images from triple-color immunofluorescent staining in P42 urethral-tube (**a**) and prostate (**b**) tissues of *Olfm4*^*eGFP*^ mice with Olfm4, Foxa1, and Sca-1 or Ck5 antibodies. UT, urethral tubes; AP, anterior prostate; DLP, dorsolateral prostate; VP, ventral prostate. Scale bars: 20 μm. Blue represents DAPI nuclear staining. (**c**, **d**) Representative single-color or merged images from triple-color immunofluorescent staining in P42 (**c**) and P56 (**d**) urethral tubes of *Olfm4*^*eGFP*^ mice with Olfm4, Ck5, and Ck4 or Ck8 antibodies. Scale bars: 20 μm. Blue represents DAPI nuclear staining.

To investigate Olfm4+ cells during the later stages of postnatal development, we performed immunofluorescent staining in P42 and P56 mouse tissues. Olfm4+ epithelial cells were observed in the urethral tubes, and these cells co-expressed Foxa1, but not Sca-1, in P42 mice (Fig. 4a). Olfm4+ epithelial cells were observed in AP, and these cells co-expressed Foxa1 in P42 mice (Fig. 4b). In contrast, Olfm4+/Foxa1+ epithelial cells were not observed in dorsolateral prostate and ventral prostate in P42 mice (Fig. 4b). Consistently, Olfm4+/Ck4- cells were observed in the AP but Ck4+/Ck8+ cells in periurethral-tube and periurethral-tube internal regions were Olfm4 negative in P56 mice (see Supplementary Fig. S4 online). In addition, Olfm4+/Syn- epithelial cells were observed in AP but not in periurethral-tube and periurethral-tube internal regions in P56 mice (see Supplementary Fig. S4 online). The Olfm4+ cells in the epithelia of urethral tubes co-expressed Ck4 and Ck8 in both P42 (Fig. 4c) and P56 (Fig. 4d) mice. Relative to the strong Olfm4 expression observed at P14, Olfm4 expression was decreased in ampullary-gland epithelium and seminal-vesicle epithelium in P42 (see Supplementary Fig. S3 online) and P56 (see Supplementary Fig. S3 online) mice. These results suggest that Olfm4+ cells persist during later development in the epithelium of urethral tube, AP and disappear in dorsolateral prostate, ventral prostate, ampullary-gland epithelium, and seminal-vesicle epithelium.

### Olfm4/eGFP+ adult mouse prostate cells formed colonies, spheres, or organoids in culture

**Figure 5:**
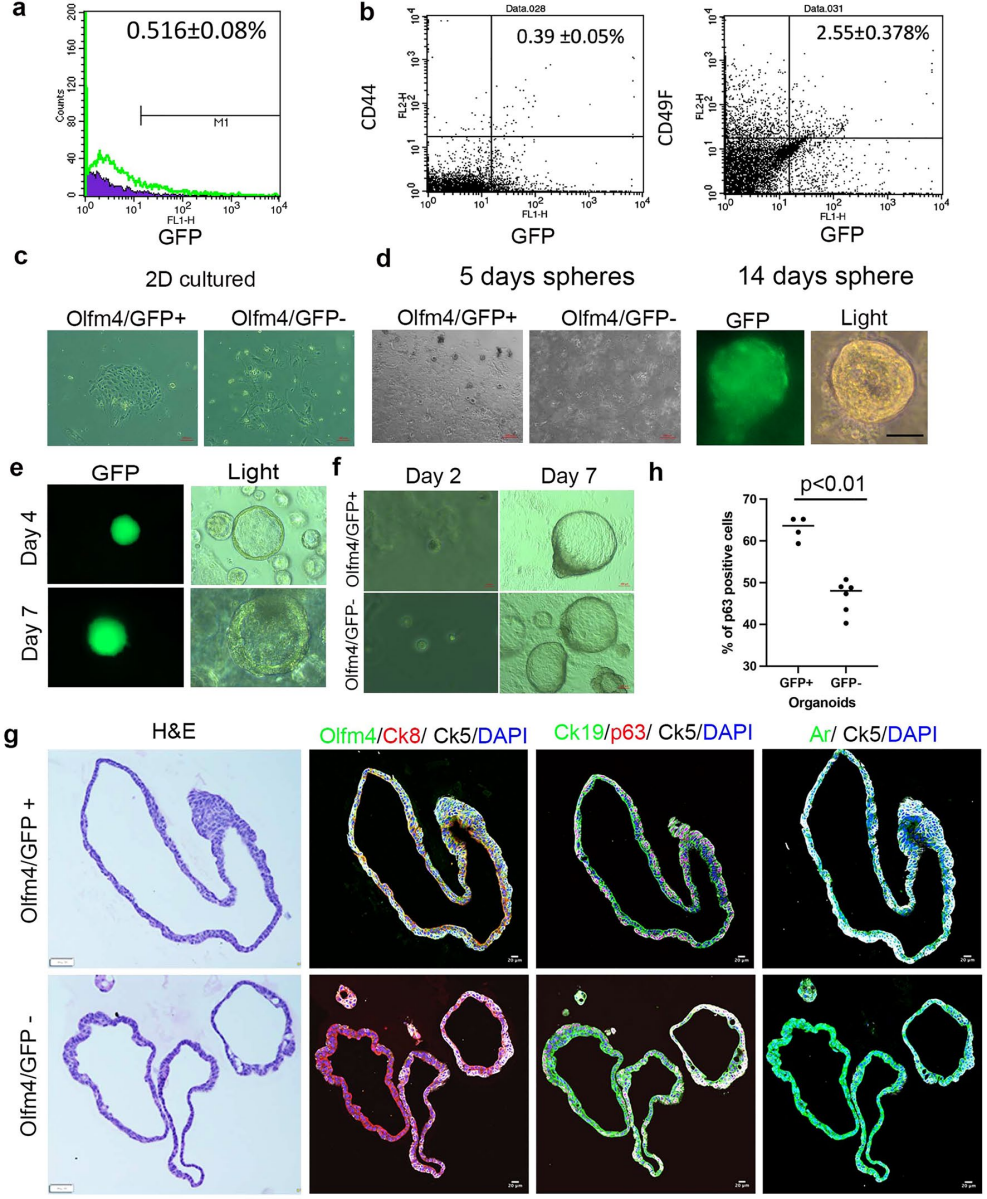
Olfm4/eGFP+ adult mouse prostate cells formed colonies, spheres, or organoids in culture. (**a**) Representative graph from FACS-based cell-sorting analysis of Olfm4/eGFP+ cells isolated from whole adult prostate from *Olfm4*^*eGFP*^ mice. Data represent mean ± SD of 3 independent experiments. (**b**) Representative graph from FACS analysis of Olfm4/eGFP/CD44+ and Olfm4/eGFP/CD49F+ cells isolated from whole adult prostate from *Olfm4*^*eGFP*^ mice. Data represent mean ± SD of 3 independent experiments. (**c**) Representative cell-growth images of Olfm4/eGFP+ or Olfm4/eGFP- cells sorted following single-cell preparation of whole adult *Olfm4*^*eGFP*^ mouse prostate. After sorting, cells were grown in 2D culture for 7 days. Experiments were repeated 3 times. Scale bars: 200 μm. (**d**) Representative cell-growth images of Olfm4/eGFP+ and Olfm4/eGFP- cells sorted following single-cell preparation of whole adult *Olfm4*^*eGFP*^ mouse prostate. After sorting, cells were subjected to prostate sphere-formation assays for 5 and 14 days. Experiments were repeated 3 times. GFP, GFP filter; Light, light field. Scale bar: 20 μm. (**e**) Representative cell-growth images of Olfm4/eGFP+ cells sorted following single-cell preparation of whole adult *Olfm4*^*eGFP*^ mouse prostate. After sorting, cells were subjected to prostatic-organoid culture for 4 and 7 days. Experiments were repeated 3 times. GFP, GFP filter; Light, light field. Scale bar: 10 μm. (**f**) Representative cell-growth images of Olfm4/eGFP+ and Olfm4/eGFP- cells sorted following single-cell preparation of whole adult *Olfm4*^*eGFP*^ mouse prostate. After sorting, cells were subjected to prostatic-organoid culture for 2 and 7 days. Experiments were repeated 3 times. Scale bars: 20 and 200 μm. (**g**) Olfm4/eGFP+ and Olfm4/eGFP- cells were sorted following single-cell preparation of whole adult *Olfm4*^*eGFP*^ mouse prostate. After sorting, cells were subjected to prostatic-organoid culture for 7 days. Left, representative images of H&E staining in Olfm4/eGFP+ cell- and Olfm4/eGFP- cell–formed organoids. Right, representative merged images from double-color or triple-color immunofluorescent staining in Olfm4/eGFP+ cell- and Olfm4/eGFP- cell–formed organoids with Olfm4, Ck8, and Ck5 antibodies, with Ck19, p63, and Ck5 antibodies, or with androgen receptor and Ck5 antibodies. Experiments were repeated 3 times. Ar, androgen receptor. Scale bars: 20 μm. Blue represents DAPI nuclear staining. (**h**) Quantitation of the p63+ cells in Olfm4/eGFP+ cell- and Olfm4/eGFP- cell–formed large organoids. Data were obtained from analysis of single-color images from immunofluorescent staining with P63 antibody using the 3D objects counter function (Image J) for each individual organoid. Olfm4/eGFP+ cell- (n = 4); Olfm4/eGFP- cell–(n = 6). *P* < 0.01 (two-tail student T test).

To further study Olfm4+ cells in the murine prostate, we performed FACS analysis and cell-culture experiments with adult *Olfm4*^*eGFP*^ mouse prostate cells. FACS analysis indicated that only approximately 0.5% of cells isolated from whole adult *Olfm4*^*eGFP*^ mouse prostate were Olfm4/eGFP+ (Fig. 5a). We have obtained approximately 0.4% of Olfm4/eGFP+/CD44+ cells and 2.5% of Olfm4/eGFP+/CD49f+ cells from whole adult *Olfm4*^*eGFP*^ mouse prostate (Fig. 5b). These results are similar with IF data (Fig. 1) indicate that Olfm4+/eGFP+ cells are heterogeneous cells population in the mouse prostate.

When Olfm4/eGFP+ and Olfm4/eGFP– cells were sorted from whole adult *Olfm4*^*eGFP*^ mouse prostate single-cell preparations and used in functional analyses in vitro*,* Olfm4/eGFP+ cells grown in 2D cultures for 7 days formed colonies composed of more than 50 epithelial cells, whereas Olfm4/eGFP– cells failed to produce colonies (Fig. 5c). Further, only Olfm4/eGFP+ cells formed spheres in 3D culture (prostate sphere-formation assays; Fig. 5d). These results indicate that Olfm4+ cells can grow in 2D and 3D prostate cell–growth culture conditions.

Next, we used Olfm4/eGFP+ cells sorted from whole adult *Olfm4*^*eGFP*^ mouse prostate in prostatic-organoid culture assays and traced single-cell growth using the GFP reporter protein to follow organoid formation for 7 days (Fig. 5e). Olfm4/eGFP+ and Olfm4/eGFP- cells formed both small and larger organoids (Fig. 5e,f). Immunofluorescent staining of organoids after 7 days of culture identified 3 cell populations within both Olfm4/GFP+ cell- and Olfm4/GFP- cell–formed larger organoids: Olfm4+/Ck8+/Ck5+ cells (Fig. 5g, see Supplementary Fig. S5A online); Ck19+/p63+ /Ck5+ basal cells (Fig. 5g); and Ck8+/androgen receptor (Ar)+ luminal cells (Fig. 5G). In contrast, only Olfm4+/Ck8+/Ck5- luminal cells were observed in Olfm4/eGFP+ cell–formed small organoids (see Supplementary Fig. S5 online). The Ck19+/p63+/Ck5+ basal-like cells were not observed in Olfm4/eGFP+ cell–formed small organoids (see Supplementary Fig. S5 online). Ar+/Ck5- luminal-like cells were observed in Olfm4/eGFP+ cell–formed small organoids (see Supplementary Fig. S5 online). Synaptophysin-positive neuroendocrine-like cells were not detected in Olfm4/eGFP+ cell–formed larger organoids (see Supplementary Fig. S5 online). To compare the difference between the larger organoids formed by Olfm4/eGFP+ and by Olfm4/eGFP- cells, we counted the P63+ cells staining in the organoid tissues. We found that Olfm4/eGFP+ cell–formed larger organoids contained a higher percentage of P63+ cells compared with Olfm4/eGPF- cell–formed larger organoids (62.9 ± 2.8% vs 46.6 ± 3.9%, respectively, *p* < 0.01 two-tail student T-test), which indicates that Olfm4/GFP+ cells prefer to be differentiated into P63+ basal cells in the organoid culture (Fig. 5h).

### Olfm4+ cells in adult mouse urethral-tube epithelium are upregulated for genes and for signaling pathways involved in development and homeostasis

**Figure 6:**
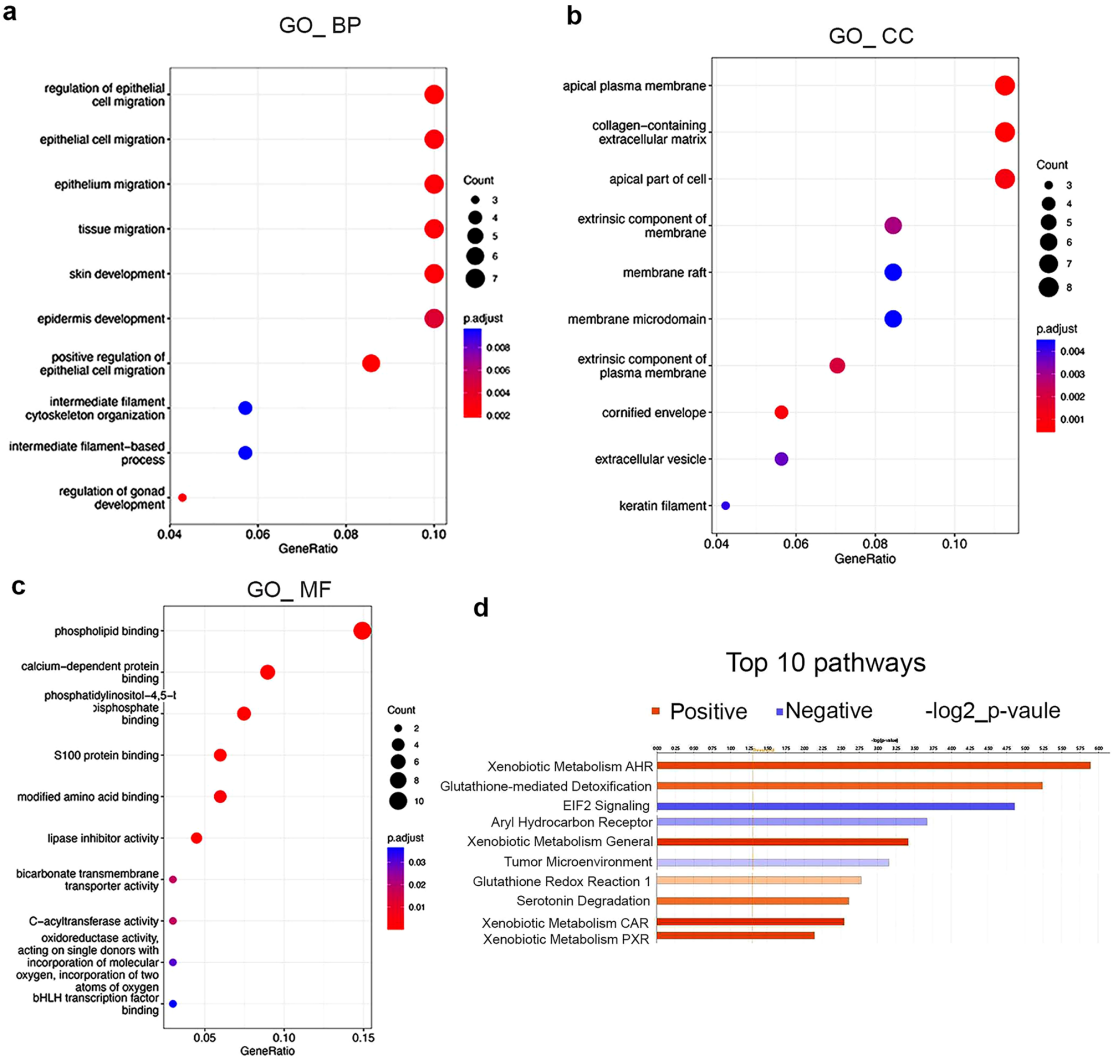
Olfm4+ cells in adult mouse urethral-tube epithelium are upregulated for genes and for signaling pathways involved in development and homeostasis. (**a**–**c**) Olfm4+ cells in GSE145865 cluster 1 ULE were subjected to Gene Ontology analysis. Bioinformatic analysis results are presented for the GO_Biological Process (GO_BP) (**a**), GO_Cellular Component (GO_CC) (**b**), and GO_Molecular Function (GO_MF) (**c**) categories. Dots represent term enrichment with color coding: red indicates high enrichment, blue indicates low enrichment. The dot size represents the percentage of each row (GO category). (**d**) Olfm4+ cells in GSE145865 cluster 1 ULE were subjected to Ingenuity Pathway analysis. Bioinformatic analysis results indicate the top 10 activated or inhibited pathways identified. Orange bars indicate positively regulated pathways; blue bars indicate negatively regulated pathways. AHR, aryl hydrocarbon receptor; EIF2, eukaryotic initiation factor 2; CAR, constitutive androstane receptor; PXR, pregnane X receptor.

To explore the biological functions of Olfm4+ cells in the epithelium of urethral tubes, we performed Gene Ontology and Ingenuity Pathway analyses using the GSE145865 dataset (single-cell RNA sequencing data from adult mouse urethra)^18^. Gene Ontology analysis of Olfm4+ cells from GSE145865 cluster 1 ULE revealed that the Biological Process category genes with the highest enrichment were related to epithelial cell migration and development (Fig. 6a). The highest enrichment in the Cellular Component category was for apical plasma membrane, collagen-containing extracellular matrix, and apical part of cell (Fig. 6b). In the Molecular Functions category, the highest enrichment was for phospholipid binding genes (Fig. 6c). Ingenuity Pathway analysis identified the top 10 pathways that were significantly positively or negatively regulated in Olfm4+ cells from GSE145865 cluster 1 ULE. Four xenobiotic metabolism signaling pathways (aryl hydrocarbon receptor [AHR], general, constitutive androstane receptor [CAR], and pregnane X receptor [PXR]) were identified as significantly positively regulated (Fig. 6d). AHR is a master transcription factor and plays important roles in detoxification, development, and immune response^20^. CAR plays a crucial role in the regulation of drug metabolism, energy homeostasis, and cancer development^21^. PXR is a key regulator of xenobiotic metabolism^22^. The glutathione-mediated detoxification, glutathione redox reaction 1, and serotonin degradation pathways were also positively regulated (Fig. 6d). The eukaryotic initiation factor 2 (EIF2) signaling, AHR, and tumor microenvironment pathways were identified as significantly negatively regulated (Fig. 6d). These results suggest that Olfm4+ epithelial cells may play an important role during development and homeostasis of the murine urethral-tube epithelium.

## Discussion

In this study, we identified that Olfm4+ cells are E-cadherin+/CD44+/Foxa1+ epithelial cells, with some of the Olfm4+ cells being a subpopulation Ck8+/Ck5+/Sca-1-/Ck4-/Syn- cells as well as Olfm4+/Ki67+ proliferative cells in the adult mouse AP epithelium. We reported a novel *Olfm4*^*eGFP*^ mouse model for tracking Olfm4/eGFP expressing cells in murine prostatic, ampullary-gland, seminal-vesicle and urethral epithelium as well as small-intestine epithelial cells during postnatal development. We have demonstrated the properties of Olfm4/eGFP+ cells with single cell that was isolated using GFP from *Olfm4*^*eGFP*^ adult mouse prostate in in vitro studies. The *Olfm4*^*eGFP*^ mouse model shed a new light for further study of Olfm4’s biological functions in murine tissues and could be used to further understand the role of OLFM4 during normal human development and disease progression.

Embryonic development studies have revealed that murine prostate derives from the urogenital sinus and prostatic epithelium buds that are initially formed at E17.5 days, then extend and differentiate throughout embryonic development^23,24^. During postnatal development from birth day 1 to day 21, prostatic epithelium buds undergo extensive ductal outgrowth and branching^24^. The distinct anterior, dorsal, lateral, and ventral lobes of the prostate are formed during the first 3 weeks of postnatal development and are completely developed by 8 to 10 weeks after birth^24,25^. Using our *Olfm4*^*eGFP*^ reporter mouse model, we observed Olfm4/eGFP expression during early postnatal development in the urogenital sinus derived epithelium, such as prostate, urethral-tube, ampullary-gland and seminal-vesicle as well as in small-intestine epithelial cells. In later postnatal development and in adult mice, we found that Olfm4 expression persisted in urethral-tube and AP epithelium but was lacking in dorsolateral and ventral prostate. These Olfm4 expression pattern indicates that spatiotemporal regulation of Olfm4 gene expression occurs during postnatal development and in adult mice. Given that multiple factors and signaling pathways that regulate Olfm4 expression in myeloid and small-intestine cells have been identified in previous studies^4^, we hypothesize that multiple factors and signaling pathways, such as AR signaling, Wnt signaling, Notch signaling, and EGFR signaling, are involved in the regulation of Olfm4-specific expression in the mouse prostate.

We found that higher populations of Olfm4+ cells are distributed in the distal region of adult mouse AP epithelium. The 38% of Olfm4+ cells were Ki67+ in the adult mouse AP epithelium, which indicates that subpopulation of Olfm4+ cells are proliferative cells. Our in vitro functional studies using single-cell preparations isolated from adult *Olfm4*^*eGFP*^ mouse prostate demonstrated that Olfm4/eGFP+ cells can grow and form spheres and organoids in culture. Importantly, we found that Olfm4/eGFP+ cell–formed larger organoids contain higher percentage of p63+ cells compared with Olfm4/eGPF- cell–formed larger organoids, which indicates that Olfm4/GFP+ cells prefer to be differentiated into P63+ basal cells in the organoid culture and that Olfm4/eGFP+ cell–formed small organoids composed only luminal lineage cells. Our findings suggest that Olfm4 is expressed in the developing epithelial cells population during postnatal development prostate and in adult mouse prostate.

When we examined urethral expression of Olfm4, we found that Olfm4 is strongly expressed in epithelial cells of the murine urethral tube from early postnatal development to adulthood. Our Gene Ontology and Ingenuity Pathway analyses of Olfm4+ cells from single-cell RNA sequencing data for luminal epithelial cells of the adult mouse urethra (GSE145865) identified upregulation of genes related to cell and tissue migration and development and the apical region of the cell, as well as upregulation of xenobiotic metabolism signaling pathways. The OLFM4 protein has previously been localized on the apical plasma membrane of cells and been determined to help form a barrier for protecting cells from the cell microenvironment in human inflamed intestinal epithelium^26^. The higher expression of Olfm4 protein may play a similar role by forming a barrier for protecting cells from the cell microenvironment in urethral-tube epithelium. Therefore, given our collective findings, the Olfm4+ urethral-tube luminal epithelium cells that we examined in this study appear to be equivalent to human urethral-tube luminal epithelium cells, and we postulate that Olfm4+ cells may function in homeostasis of urethral-tube epithelium during postnatal development and in adult mice.

It has been reported that OLFM4+ cells in the urethral luminal epithelium were increased in human benign prostatic hyperplasia (BPH) and survive treatment with 5-a-reductase inhibitor (5ARI), which inhibits the conversion of testosterone to dihydrotestosterone^27^. Because of findings that OLFM4 is expressed in both mouse and human urethral luminal epithelial cells, Olfm4 mouse model may be useful for BPH preclinical studies. Furthermore, it is widely recognized that tumor cells hijack early development mechanisms to drive their uncontrolled proliferation and progression^28^. We have previously reported that aging *Olfm4* knockout mice sporadically developed prostatic neoplasia^12^. However, the cells of origin that became malignant in these mice have not yet been identified. We will further study whether Olfm4+ cells are the cells of origin during murine prostatic carcinogenesis. The *Olfm4*^*eGFP*^ reporter mouse model described here provides a novel tool for studying biological functions of Olfm4 in murine tissues and could be used to further understand the role of OLFM4+ cells during normal human development and disease progression, including in prostatic diseases such as BPH and prostate cancer.

## Methods

### Mouse experiments

All mouse experiment protocols were approved by the Animal Care and Use Committee of the National Heart, Lung, and Blood Institute (NHLBI). Animal care was performed in accordance with relevant institutional and national guidelines and regulations in the animal facilities of the National Institutes of Health (NIH). The study is reported in accordance with “ARRIVE guidelines”.

### Generation of ***Olfm4***^***eGFP***^ reporter mice

To generate *Olfm4*^*eGFP*^ mice, we constructed a 14.4 kb targeting vector (Fig. 2a). To prepare the targeting vector, 2.03 and 5.04 kb fragments flanking the *Olfm4* gene were cloned by PCR from genomic DNA of R1 ES cells to serve as 5′ and 3′ homologous arms. The *EGFP-2A* gene was fused in‐frame to the ATG at the *Olfm4* translation start site in the 5′ homologous arm. The resulting 5′ and 3′ arms were inserted into 5′ and 3′ multiple‐cloning sites of the pKS‐TK‐Neo‐LoxP vector (Ingenious Targeting Laboratory). Targeted ES cell clones were obtained by homologous recombination in R1 ES cells. The neomycin resistance gene was deleted by transient transfection of the Cre recombinase expression vector pPAC‐Cre (Ingenious Targeting Laboratory).

A 7.64-kb genomic DNA used to construct the targeting vector was first subcloned from a positively identified B6 BAC clone (RP23: 162K11). The targeting vector was confirmed by restriction analysis and sequencing after each modification. The boundaries of the 2 homology arms were confirmed by sequencing with P6 and T73 primers (see Supplementary Table S1 online) that read through both sides of the backbone vector into the genomic sequence. The FRT-Neo-FRT cassette was confirmed by sequencing with LAN1 and iNeoN2 primers (see Supplementary Table S1 online) that read from the 5′ and 3′ ends of the Neo cassette, respectively, into the genomic sequences. The *Olfm4* and *eGFP-2A* sequence was sequencing-confirmed with OLFMSQ1, OLFMSQ2, and FALOGFP3 primers (see Supplementary Table S1 online).

The BAC was subcloned into a ~ 2.4-kb pSP72 (Promega) backbone vector containing an ampicillin selection cassette for retransformation of the construct prior to electroporation. A pGK-gb2 FRT-flanked Neo cassette was inserted into the gene (Fig. 2a). The targeting vector was linearized using NotI and then transfected into BA1 (129/SvEv x C57BL/6) (Hybrid) ES cells by electroporation. After selection with G418 antibiotic, surviving clones were expanded and subjected to PCR analysis to identify recombinant ES clones containing the GFP cassette. PCR was performed using the OLFM SC1 and UNI primers (see Supplementary Table S1 online). Sequencing was performed on purified PCR products to confirm the presence of the genomic/5′ cassette junction using the OLFM SC1 primer, and to confirm the presence of the 3′ cassette sequences/genomic junction using the GFP SC1 primer (see Supplementary Table S1 online).

Positive clones identified by PCR were subjected to Southern-blotting analysis to confirm integration of the targeting vector. DNA was isolated, digested with MfeI or NsiI, and electrophoretically separated on a 0.8% agarose gel. After transfer to a nylon membrane, the digested DNA was hybridized with a probe (iNeo) targeted against the Neo cassette (see Supplementary Table S1 online).

Targeted ES cells were injected into blastocysts of C57BL/6 mice to obtain chimeric founders. The obtained *Olfm4*^*eGFP/*+^ heterozygotes were backcrossed with C57BL/6 mice. Mice were bred and maintained at the National Heart, Lung, and Blood Institute (NHLBI)/National Institutes of Health (NIH) under protocol H-0226.

### *Olfm4* knockout mice

Generation of *Olfm4* knockout mice has been described previously^10^. *Olfm4* knockout mice were maintained by crossing *Olfm4*(+ /−) mice. Mice were genotyped using PCR and primers (see Supplementary Table S1 online) as described previously^10^. The genetic background of these mice was C57BL/6.

### PCR genotyping

Mouse genomic DNA was extracted by using QIAGEN’s DNA extraction kit following the manufacturer’s instructions. PCR was conducted using the NDEL primer set (see Supplementary Table S1 online) to screen for the *Olfm4*^*eGFP*^ allele with deletion of the Neo cassette. PCR conditions were an initial 2 min hot start at 94 °C, followed by 30 cycles at 94 °C (30 s), 60 °C (30 s), and 70 °C (1 min). PCR products were run on a 2% agarose gel with a 100-bp ladder as reference. The PCR product size for the wild-type *Olfm4* allele is 289 bp and for the *Olfm4*^*eGFP*^ allele is 426 bp due to the Neo cassette deletion (because 1 set of LoxP-FRT sites [137 bp] remains).

### Mouse tissue collection and immunohistochemical and fluorescent immunohistochemical staining

Prostate microdissection was performed following a previously described procedure^29^; other tissues were also harvested at the time of sacrifice by using Forane (NDC-10019-360-60, Baxter) following the animal protocol (NHLBI-H-0226). Immunohistochemical analyses were performed on formalin-fixed paraffin sections as previously described^7^. Immunohistochemical staining was performed with antibodies listed in Supplementary Table S2 online. Secondary antibodies, Super Sensitive MultiLink, and Super Sensitive Label were purchased from BioGenex. Dark brown color was developed with chromagen (BioGenex). Sections were counterstained with hematoxylin.

All images of immunohistochemical staining were acquired using an Olympus BX51 microscope (Olympus) and Qimaging Camera with Q Capture pro software (Qimaging). Images were acquired using the × 40 (air) or × 60 Uplan Apo objective (1.42 oil), then imported into Adobe Photoshop for presentation. All experiments were performed with 3 mouse tissue samples and repeated 3 times with *Olfm4*+*/*+, *Olfm4-/-*, and *Olfm4*^*eGFP*^ mice.

Fluorescent immunohistochemistry was performed on unstained paraffin sections of murine prostate and other tissues as described previously^8^. Images were obtained using the ZEISS 880 Confocal Microscope (inverted). Primary and secondary antibodies used for staining are listed in Supplementary Table S2 online. The images were processed with Fiji 3 software for 2D images. The combined picture panels were assembled with Adobe Photoshop CC 2017. All experiments were performed with 3 mouse tissue samples and repeated 3 times with *Olfm4*+*/*+ and *Olfm4*^*eGFP*^ mice.

### Quantitation of positive staining cells

For quantitation of Olfm4+ cells or Ki67+ cells in adult mouse AP, we captured 5–10 fluorescent immunohistochemistry staining images using the ZEISS 880 Confocal Microscope (inverted) with 40 oil objectives from each individual adult mouse tissues. The images were processed with Fiji III software (NIH). Quantitation of the Olfm4+ or Ki67+ cells was performed from single-color images or merged images from immunofluorescent staining with Olfm4 and Ki67 antibodies within the AP of *Olfm4* wild-type adult mice obtained using the 3D objects counter function (Image J). The cells were counted from a total of 5–10 images for each individual mouse. For quantitation of P63 positive cells, we counted the single-color images from immunofluorescent staining with P63 antibody using the 3D objects counter function (Image J) for each individual organoid. Total number of cells within the images were counted with DAPI single-color nuclei staining by using the 3D objects counter function (Image J).

### Cell sorting and FACS analysis

Mouse prostate tissue was processed to prepare a single-cell suspension following a protocol described previously^30^. Briefly, prostate tissues from 8-week-old *Olfm4*^*eGFP*^ mice were combined, digested into single cells, and sorted with GFP using FACS. For cell sorting, 1–2 × 10^7^ cells/ml were washed once with medium, then resuspended in 1 ml medium. Anti-GFP Alexa Fluor 488-conjugated antibody (1:200; Invitrogen) was added to the cell suspension and incubated on ice for 30 min. After washing and filtering with a cell strainer (100-µm filter, BD Falcon™), FACS-based cell sorting was performed with BD Aria (BD Biosciences). For FACS analysis, 1–2 × 10^6^ cells/ml were resuspended in 1 ml PBS and stained with anti-GFP Alexa Fluor 488-conjugated antibody (1:200) and anti-CD44 phycoerythrin-conjugated antibody (1:200, eBioscience) for 1 h at room temperature, mixing with rotation. The cells were then washed with PBS once and resuspended in 0.5 ml PBS, then analyzed by flow cytometry (BD FACSCalibur, BD Biosciences). The experiments were repeated 3 times.

### 2D cell cultures

Olfm4/eGFP-positive and -negative cells were sorted with GFP using FACS as described above. Sorted cells were seeded into 6-well plates (1 × 10^4^ cells/well) and grown in prostate epithelial cell growth medium^30^. Cells were photographed at day 7 under both light (AX10 Cam 503 mono) and GFP (AX10 Cam 105 Color) conditions with a ZEISS microscope (AX 10) and ZEISS software. All images were processed with Adobe Photoshop for presentation. The experiments were repeated 3 times.

### Prostate sphere-formation assay

Prostate sphere-formation assays were performed following a previously described protocol^30^. Olfm4/eGFP-positive and -negative cells were sorted with GFP using FACS as described above. Cells (1 × 10^4^) were suspended in 50 μl growth medium and mixed with 50 μl Matrigel (BD Biosciences), then cultured in 12-well plates for up to 14 days. Images of spheres were captured with an AX10 Cam 503 mono or GFP AX10 Cam 105 Color with a ZEISS microscope (AX 10) and ZEISS software. All images were processed with Adobe Photoshop for presentation. The experiments were repeated 3 times.

### Prostatic-organoid culture

Organoid culture was performed following the protocol published previously by Drost et al.^31^. Olfm4/eGFP-positive and -negative cells were sorted with GFP using FACS as described above. Sorted GFP+ cells (4 × 10^5^) were mixed with 40 μl Matrigel and then placed in the center of each well in a 24-well plate. Murine organoid culture medium (0.5 ml) was then added to each well. Organoid growth was traced from day 1 to day 7 by taking pictures of GFP+ single cells using a ZEISS AXIO microscope with either a GFP filter or using a light field. Organoid images were processed with Adobe Photoshop software. For tissue analysis, organoids that had been cultured for 7 days were fixed with 10% formalin solution in PBS at room temperature for 1 h, then changed into 70% ethanol overnight. After paraffin embedding, 5-μm sections of organoids were cut, and paraffin section slides used for H&E staining and fluorescent immunohistochemistry. H&E staining was performed on organoids using standard procedures. The experiments were repeated 3 times.

### Single-cell RNA-sequencing meta data processing and gene ontology analysis

Raw counts as deposited in GEO (accession ID GSE145865: single-cell RNA sequencing data from adult mouse urethra^18^) were downloaded and analyzed using version 4 of Seurat R package^32^. For processing, we removed cells that had greater than 5% of mitochondrial reads and less than 400 genes expressed. To mitigate doublets, driven by basic quality control violin plots we removed cells that had greater than 1500 (sample 1) and 4500 (sample 2) genes expressed. We also filtered cells that had greater than 5000 (sample 1) and 20,000 (sample 2) reads that aligned to the transcriptome. For normalization, we used the scTransform function in Seurat. After integrating the samples, we used 30 principal components to perform dimensionality reduction and identify clusters using a resolution parameter of 0.2. For Gene Ontology analysis of Olfm4+ cells in GSE145865 cluster 1 ULE, we used the enrichGO and simplify functions from the ClusterProfiler R package^33^.

## Supplementary Information

Supplementary Information.

## Data Availability

All data generated or analyzed during this study are included in this published article and its supplementary information files.
