# Supplementary material for: Characterization of olfactomedin 4+ cells in prostate and urethral-tube epithelium during murine postnatal development and in adult mice
Source: Sci Rep. 2023 Jun 25;13:10290. doi: 10.1038/s41598-023-37320-9 (PMC10290983; doi:10.1038/s41598-023-37320-9)
Supplement: Supplementary file 1 — Supplementary Information. [file 41598_2023_37320_MOESM1_ESM.pdf]

Characterization of olfactomedin 4+ cells in prostate and urethral-tube epithelium during murine postnatal development and in adult mice

Hongzhen Li[1], Vijender Chaitankar[2], Lena Cui[3], Weiping Chen[3], Kyung Chin[1], Jianqiong Zhu[1], Wenli Liu[1], and Griffin P. Rodgers[1]

[1]Molecular and Clinical Hematology Branch, National Heart, Lung, and Blood Institute, National Institutes of Health, Bethesda, MD 20892, USA

[2]Bioinformatics and Systems Biology Core, National Heart, Lung, and Blood Institute, National Institutes of Health, Bethesda, MD 20892, USA

[3]Genomics Core, National Institute of Diabetes and Digestive and Kidney Diseases, National Institutes of Health, Bethesda, MD 20892, USA

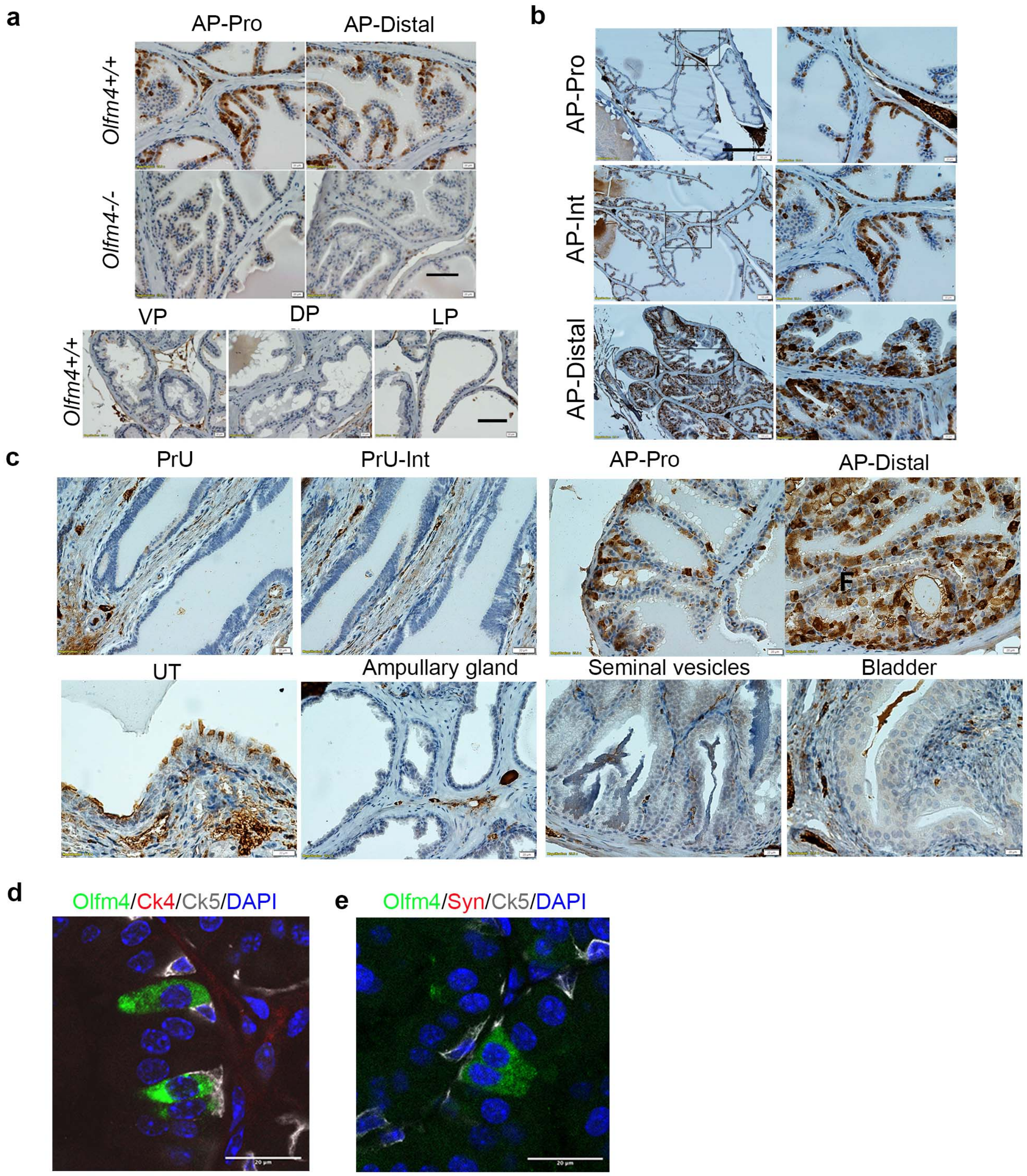

Supplementary Figure S1. Olfm4 is strongly expressed in epithelial cells of anterior prostate and urethral tube of adult mice. (**a**) Representative images of immunohistochemical staining with Olfm4 antibody in prostate of *Olfm4* wild-type (*Olfm4+/+*) and *Olfm4* knockout (*Olfm4–/–)* adult mice. AP-Pro, proximal anterior prostate; AP-Distal, distal anterior prostate; VP, ventral prostate; DP, dorsal prostate; LP, lateral prostate. Scale bar: 50 μm. (**b**)

Representative images of immunohistochemical staining with Olfm4 antibody in anterior prostate (AP) of *Olfm4* wild-type adult mice. Right 3 panels are enlarged images from areas of 3 left panels marked with a square. Pro, proximal; Int, internal. Scale bar: 200 μm. (**c**) Representative images of immunohistochemical staining with Olfm4 antibody in periurethral tubes (PrU), periurethral tubes internal (PrU-Int), proximal anterior prostate (AP-Pro), distal anterior prostate (AP-Distal), urethral tubes (UT), ampullary gland, seminal vesicles, and bladder of *Olfm4* wild-type adult mice. Scale bars: 20 μm. (**d, e**) Representative merged images from triple-color immunofluorescent staining in AP of *Olfm4* wild-type adult mice with Olfm4, Ck4 or synaptophysin (Syn) and Ck5 antibodies. Scale bars: 20 μm. Blue represents DAPI nuclear staining.

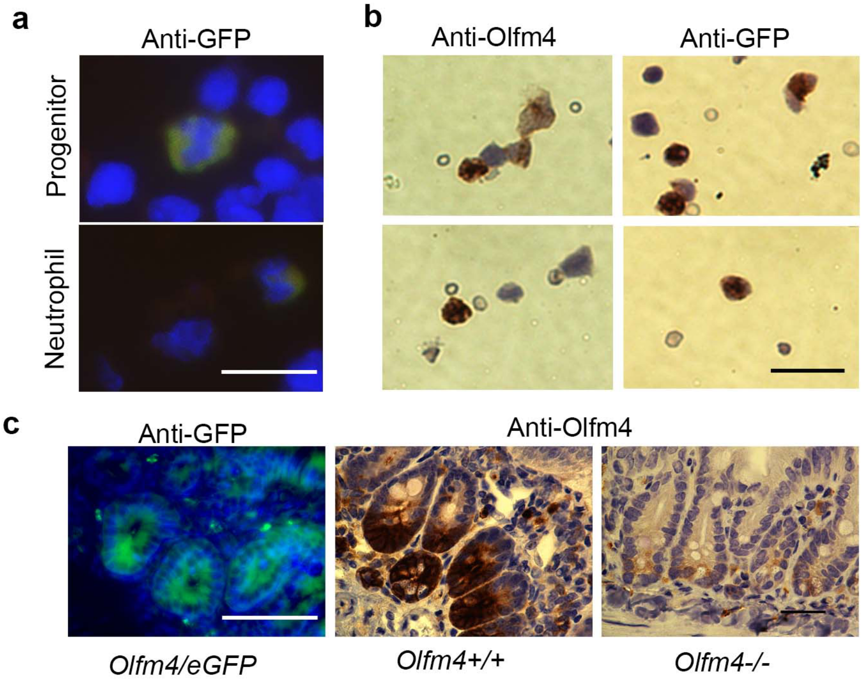

Supplementary Figure S2. Olfm4/eGFP is expressed in bone marrow and small-intestine cells in the adult *Olfm4*^{eGFP} mouse. (**a**) Representative images of immunofluorescent staining with GFP antibody (#2956, Cell Signaling Technology) in bone-marrow smear cells from adult *Olfm4*^{eGFP} mice. Blue represents DAPI nuclear staining. Scale bar: 50 µm. (**b**) Representative images of immunohistochemical staining with Olfm4 or GFP antibody in bone-marrow smear cells from adult *Olfm4*^{eGFP} mice. Scale bar: 50 µm. (**c**) Representative images of immunofluorescent staining with GFP antibody (#2956, Cell Signaling Technology) (left panel) in intestine of adult *Olfm4*^{eGFP} mice and of immunohistochemical staining with Olfm4 antibody (right 2 panels) in intestine of adult *Olfm4* wild-type (*Olfm4*+/+) and *Olfm4* knockout (*Olfm4*-/-) mice. Blue represents DAPI nuclear staining. Scale bars: 50 µm.

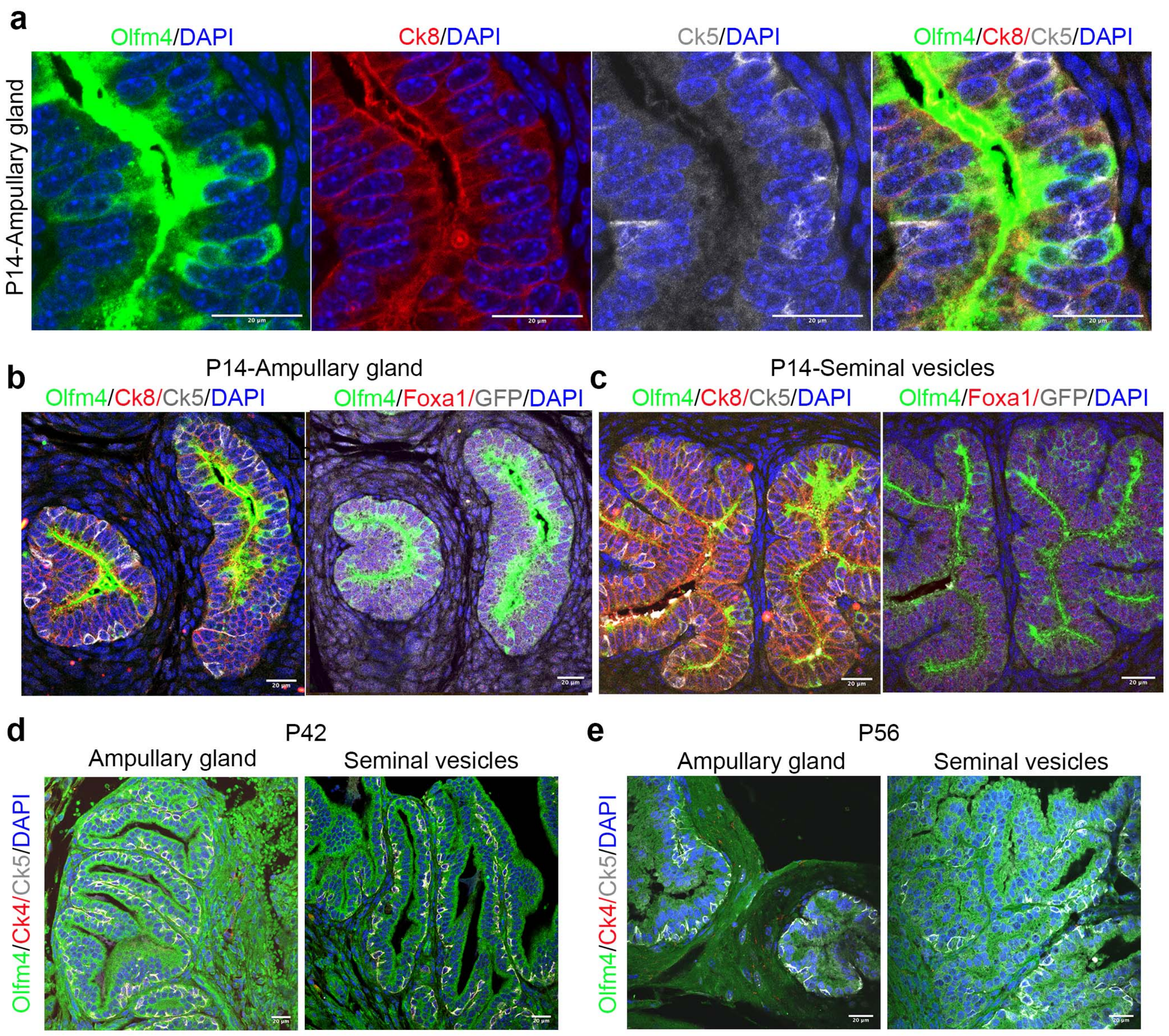

Supplementary Figure S3. Olfm4/eGFP is transientlyexpressed during postnatal development of the epithelium in *Olfm4*^{eGFP} mouse ampullary glands and seminal vesicles. (**a**) Representative single-color or merged images from triple-color immunofluorescent staining in ampullary-gland tissues of P14 *Olfm4*^{eGFP} mice with Olfm4, Ck8, or Ck5 antibody. Scale bars: 20 µm. Blue represents DAPI nuclear staining. (**b, c**) Representative merged images from triple-color immunofluorescent staining in ampullary-gland (**b**) or seminal-vesicle (**c**) tissues of P14 *Olfm4*^{eGFP} mice with Olfm4, Ck8, and Ck5 antibodies (left panel) or Olfm4, Foxa1, and GFP

(ab13970, Abcam) antibodies (right panel). Scale bars: 20 μm. Blue represents DAPI nuclear staining. (**d, e**) Representative merged images from triple-color immunofluorescent staining in ampullary-gland and seminal-vesicle tissues of P42 (**d**) and P56 (**e**) *Olfm4*^{eGFP} mice with Olfm4, Ck4, and Ck5 antibodies. Scale bars: 20 μm. Blue represents DAPI nuclear staining.

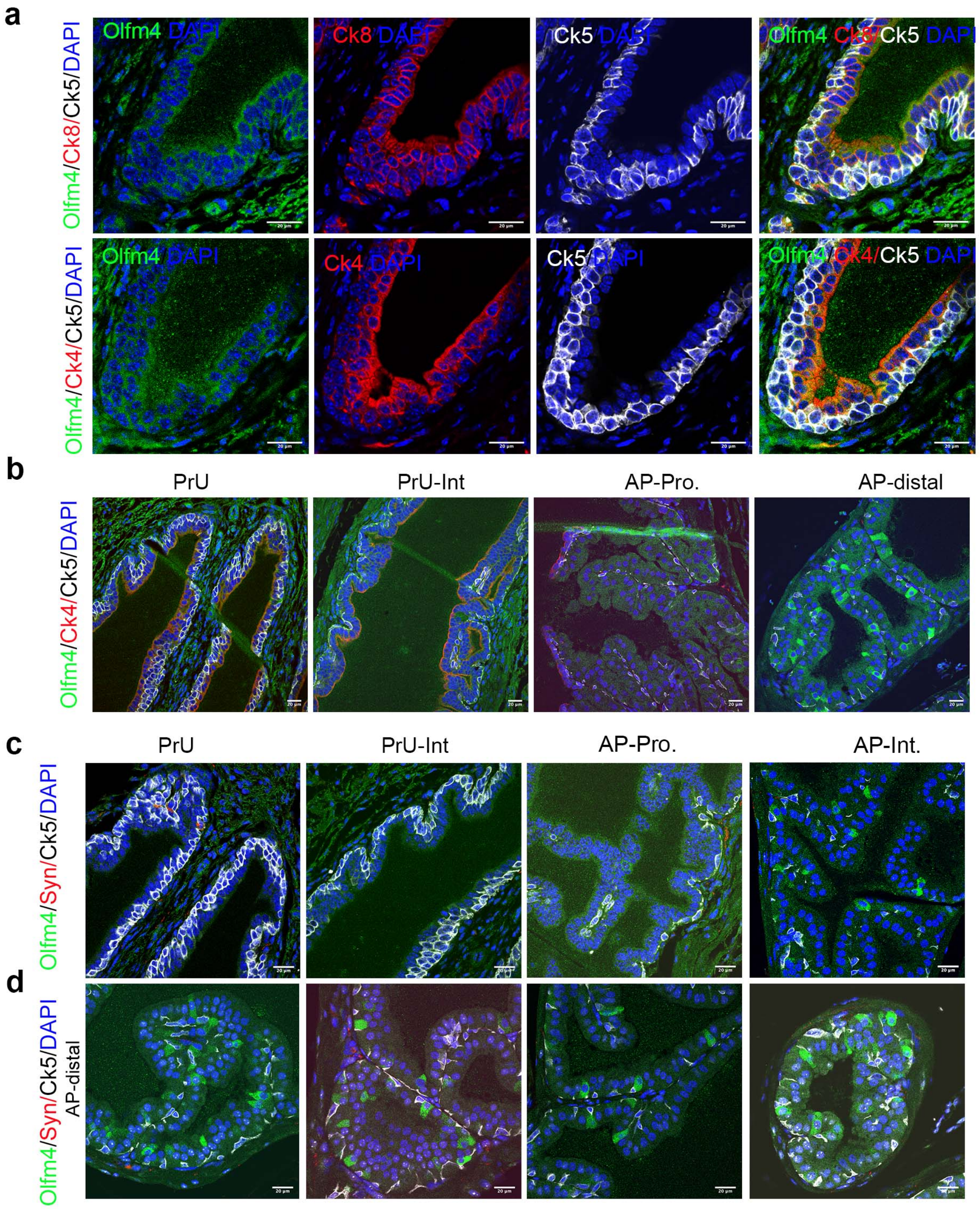

Supplementary Figure 4. Olfm4+/Syn- cells persist in the epithelium of P56 anterior prostate of *Olfm4*^{eGFP} mice. (**a**) Representative single and merged images from triple-color immunofluorescent staining in periurethral tubes (PrU) of P56 *Olfm4*^{eGFP} mice with Olfm4, Ck4, and Ck5 antibodies. Scale bars: 20 μm. Blue represents DAPI nuclear staining. (**b**)

Representative merged images from triple-color immunofluorescent staining in periurethral tubes (PrU), periurethral tubes internal (PrU-Int), proximal anterior prostate (AP-Pro), and internal anterior prostate (AP-Int) and distal of P56 *Olfm4*^{eGFP} mice with Olfm4, Ck4 and Ck5 antibodies. Scale bars: 20 µm. Blue represents DAPI nuclear staining. (**c**)Representative merged images from triple-color immunofluorescent staining in periurethral tubes (PrU), periurethral tubes internal (PrU-Int), proximal anterior prostate (AP-Pro), and internal anterior prostate (AP-Int) of P56 *Olfm4*^{eGFP} mice with Olfm4, synaptophysin (Syn), and Ck5 antibodies. Scale bars: 20 µm. Blue represents DAPI nuclear staining. (**d**) Representative merged images from triple-color immunofluorescent staining in distal anterior prostate tissues of P56 *Olfm4*^{eGFP} mice with Olfm4, synaptophysin (Syn), and Ck5 antibodies. Scale bars: 20 µm. AP-Distal, distal anterior prostate. Blue represents DAPI nuclear staining.

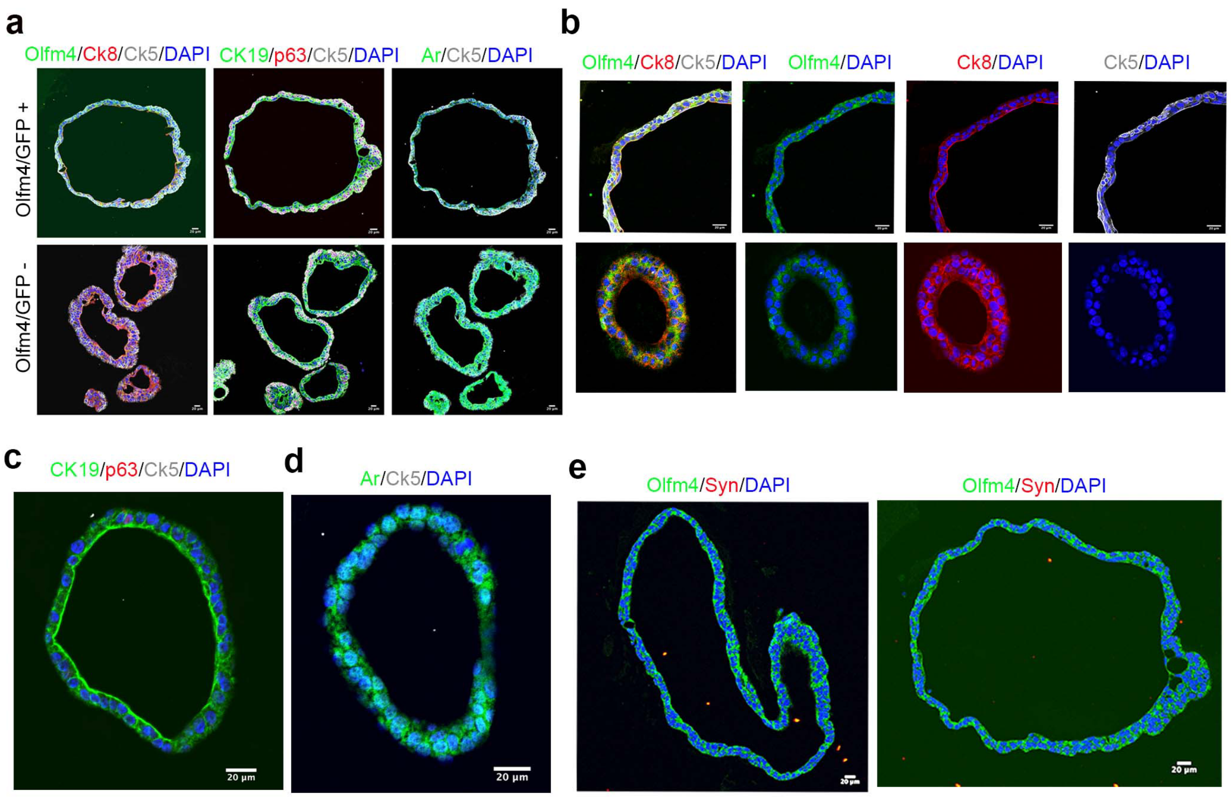

Supplementary Figure S5. Olfm4/eGFP+ adult mouse prostate cells formed organoids in culture. Olfm4/eGFP+ and Olfm4/eGFP- cells were sorted following single-cell preparation of whole adult *Olfm4*^{eGFP} mouse prostate. After sorting, cells were subjected to prostatic-organoid culture for 7 days. (**a**) Representative merged images from double-color or triple-color immunofluorescent staining in Olfm4/eGFP+ cell– and Olfm4/eGFP- cell–formed organoids with Olfm4, Ck8, and Ck5 antibodies, with Ck19, p63, and Ck5 antibodies, or with androgen receptor and Ck5 antibodies. Experiments were repeated 3 times. Ar, androgen receptor. Scale bars: 20 µm. Blue represents DAPI nuclear staining. (**b**) Representative single-color or merged images from triple-color immunofluorescent staining of Olfm4/eGFP+ cell–formed larger (upper row) and small (lower row) organoids with Olfm4, Ck8, and Ck5 antibodies. Scale bars: 20 µm. Blue represents DAPI nuclear staining. (**c–e**) Representative merged images from double-color

or triple-color immunofluorescent staining of Olfm4/eGFP+ cell–formed small organoids with Ck19, p63, and Ck5 antibodies (**c**), with androgen receptor and Ck5 antibodies (**d**), or with Olfm4 and synaptophysin antibodies (**e**). Ar, androgen receptor. Syn, synaptophysin. Scale bars: 20 μm. Blue represents DAPI nuclear staining.

Supplementary Table S1. List of primers and probes used in this study.

**Primers used for sequencing**	
Primer P6	5′–GAG TGC ACC ATA TGG ACA TAT TGT C–3′
Primer T73	5′–TAA TGC AGG TTA ACC TGG CTT ATC G–3′
Primer LAN1	5′–CCA GAG GCC ACT TGT GTA GC–3′
Primer iNeoN2	5′–AGT ATG GCT TTC CTT CCC GAT GG–3′
Primer OLFMSQ1	5′–ACC TTC TGC CCA AGA AGT GCC TC–3′
Primer OLFMSQ2	5′–TGA GCC CAT ACC ACT CTC TCT GG–3′
Primer FALOGFP3	5′–CAC AAG TTC AGC GTG TCC GGC–3′
**Primers for PCR screening**	
OLFM SC1	5′–CAC AGA GTG GGG CTT GGT GCA AGG–3′
GFP SC1	5′–AGG TGA ACT TCA AGA TCC GCC ACA–3′
UNI	5′–AGC GCA TCG CCT TCT ATC GCC TTC–3′
**Primers for mouse genotyping**	
NDEL1	5′–ATG TTC TAA AGA GGT TCT CGA CCC AGG–3′
NDEL2	5′–ACT AGG TGC AGA GGA ACT GTC AAG G–3′
**iNeo probe sequence (359 bp)**	
	GTGAGTTGTCAGGTGATCCAGGAAGAGACCTTCT<br>GCAATCCAGTGACCAATTAATTACAGCAGAAAGG<br>ACCATCGGGAAGGAAAGCCATACTCTCCAGGAAC<br>GTCATTAGTCGGGATCTTCAGTTGCTACAAGAAGC<br>AGATGTCAAACGGCCTTCCCCTAACCATGTGAGA<br>AGTGAGCTTTCACTGGCCCGGGTGTGAAGTGATTC<br>TAATGGAATAAATGGATTTGCTAAGGAATAGTTTC<br>CTCAGAAGAAATCCTGGGAGCAAGTGGGGAAAGC<br>TGACTCAGCAAAACAGAGCTGTTTCTTGAGGACG<br>ATGCCAATAGCAATCATTTGACCAAACTGAAGTG<br>GCCGTCAGGAGGCATG

Supplementary Table S2. List of antibodies used in this study.

Primary Antibodies	Vendor and Cat#, clone, lot	Species
**Immunohistochemical staining**		
anti-Olfm4 (D6Y5A)	Cell Signaling Technology Inc (39141S)	Rabbit mAb
anti-GFP	Cell Signaling Technology Inc (2956)	Rabbit
**Tissue fluorescent immunohistochemistry**		
anti-GFP	Cell Signaling Technology Inc (2956)	Rabbit
anti-GFP	Abcam (ab13970)	Chicken pAb
anti-Olfm4 (D6Y5A)	Cell Signaling Technology Inc (39141S)	Rabbit mAb
anti-KRT5	Biolegend (905901)	Chicken
anti-CD44	Novus Biologicals (NBP1-47386, 8E2F3)	Mouse
anti-Foxa1	Abcam (ab55178, Lot# GR3241742-2)	Mouse
anti-Ck8	Covance (MMS-162p-250, 1E8)	Mouse
anti-Ck17/19 (D4G2)	Cell Signaling Technology Inc (12434)	Rabbit
anti-synaptophysin (SY38)	Abcam (ab8049-1)	Mouse
anti-p63 (4A4)	Santa Cruz Biotechnology, Inc (sc-8431)	Mouse
anti-Ki67	Invitrogen (14-5698-82)	Rat
anti-Ck4 (6B10)	Santa Cruz Biotechnology, Inc (sc-52321)	Mouse
anti-E-cadherin	BD Transduction Laboratories (610182)	Mouse
anti-Sca-1/Ly-6A/E (D7)	Invitrogen (REF MA1-70082)	Rat IgG
anti-androgen receptor	Abcam (ab133273)	Rabbit mAb
**Secondary Antibodies for Tissue Fluorescent Immunohistochemistry**		
Alexa Fluor^{TM} 488 goat anti-rabbit IgG (H+L)	Invitrogen (A11034)	Goat IgG
Alexa Fluor^{TM} 488 goat anti-mouse IgG (H+L)	Invitrogen (A11032)	Goat IgG
Alexa Fluor^{TM} 594 goat anti-rat IgG (H+L)	Invitrogen (A11007)	Goat IgG
Alexa Fluor^{TM} 647 goat anti-rat IgG (H+L)	Invitrogen (A21247)	Goat IgG
Alexa Fluor^{TM} 647 donkey anti-chicken IgG (H+L)	Jackson ImmunoResearch (703-606-155)	Donkey IgG
